# Vitamin D and Cardiovascular Disease

**DOI:** 10.3390/nu5083005

**Published:** 2013-07-31

**Authors:** Katharina Kienreich, Andreas Tomaschitz, Nicolas Verheyen, Thomas Pieber, Martin Gaksch, Martin R. Grübler, Stefan Pilz

**Affiliations:** 1 Department of Internal Medicine, Division of Endocrinology and Metabolism, Medical University of Graz, 8010 Graz, Austria; E-Mails: thomas.pieber@medunigraz.at (T.P.); martin.gaksch@medunigraz.at (M.G.); martin.gruebler@stud.medunigraz.at (M.R.G.); stefan.pilz@chello.at (S.P.); 2 Specialist Clinic Bad Aussee, 8990 Bad Aussee, Austria; E-Mail: andreas.tomaschitz@gmx.at; 3 Department of Cardiology, Medical University of Graz, 8010 Graz, Austria; E-Mail: nicolas.verheyen@medunigraz.at; 4 Department of Epidemiology and Biostatistics, EMGO Institute for Health and Care Research, VU University Medical Center, 1081 BT Amsterdam, The Netherlands

**Keywords:** vitamin D, 25-OH-cholecalciferol, vitamin D deficiency, cardiovascular disease, cardiovascular risk factors, mortality

## Abstract

Vitamin D deficiency, as well as cardiovascular diseases (CVD) and related risk factors are highly prevalent worldwide and frequently co-occur. Vitamin D has long been known to be an essential part of bone metabolism, although recent evidence suggests that vitamin D plays a key role in the pathophysiology of other diseases, including CVD, as well. In this review, we aim to summarize the most recent data on the involvement of vitamin D deficiency in the development of major cardiovascular risk factors: hypertension, obesity and dyslipidemia, type 2 diabetes, chronic kidney disease and endothelial dysfunction. In addition, we outline the most recent observational, as well as interventional data on the influence of vitamin D on CVD. Since it is still an unresolved issue whether vitamin D deficiency is causally involved in the pathogenesis of CVD, data from randomized controlled trials (RCTs) designed to assess the impact of vitamin D supplementation on cardiovascular outcomes are awaited with anticipation. At present, we can only conclude that vitamin D deficiency is an independent cardiovascular risk factor, but whether vitamin D supplementation can significantly improve cardiovascular outcomes is still largely unknown.

## 1. Introduction

Vitamin D is classically known for its role in bone metabolism, being important for the maintenance of calcium homeostasis by ensuring physiologic calcium absorption by the gut [1,2,3]. The discovery that the vitamin D receptor (VDR) is ubiquitously expressed in almost all body cells, such as immune, vascular or myocardial cells, suggests an involvement of vitamin D-mediated effects in several other systems apart from musculoskeletal tissues [2]. This has led to extensive research on vitamin D as a potential influencing factor in the pathogenesis of several chronic non-skeletal diseases, such as infectious or autoimmune diseases, cancer or cardiovascular diseases (CVD) [4,5,6].

Cardiovascular (CV) risk factors, such as arterial hypertension, obesity, dyslipidemia or diabetes mellitus, as well as CVDs, including myocardial infarction, coronary artery disease or stroke, are the most prevalent diseases and account for the major causes of death worldwide, especially in Western countries [7]. This underlines the importance of clarifying the role of vitamin D in the context of CVD.

Already in 1981, Scragg reported on a seasonal variation of CV mortality and suspected a positive, protective effect of UVB-radiation on CV risk [8]. An association of vitamin D and different CV risk factors and diseases has been extensively evaluated during the last few years. Numerous observational studies, prospective meta-analyses, as well as some interventional studies have addressed the possible linkage of vitamin D deficiency and the development of CVD and its risk factors [9,10,11,12].

The scope of this review is to provide a brief overview on basic vitamin D metabolism and vitamin D deficiency. We summarize the most recent studies evaluating the relationship between vitamin D and the presence of cardiovascular risk factors, including hypertension, obesity, type 2 diabetes mellitus, chronic kidney disease, dyslipidemia and endothelial dysfunction. In addition, we give an overview on observational data on the association between vitamin D status and incident CV events. Finally, we discuss randomized controlled trials (RCTs) and meta-analyses on vitamin D treatment and its influence on CVD. Since there has been extensive research, including numerous reviews on this topic within the last few years, we mainly want to concentrate on the latest developments within the years 2012 to 2013. We conclude our work by giving an outlook on expectations concerning the large ongoing interventional trials on vitamin D supplementation and CVD and the future developments in this research field.

### 1.1. Basic Vitamin D Metabolism

Vitamin D_3_ is a steroid pro-hormone, which is mainly derived from UVB-induced synthesis of 7-dehydrocholesterole in the skin. This endogenous synthesis is the main source of vitamin D supply to the body and accounts for approximately 80% of the vitamin D supply [1,2,3]. Vitamin D_2_ or D_3_ can also be taken up via nutrition in small amounts, as it is also contained in, e.g., eggs, mushrooms and fish. After synthesis in the skin or nutritional uptake, vitamin D is then transported to the liver by a specific vitamin D binding protein (VDBP), where it is hydroxylated to 25-hydroxy-vitamin D (25(OH)D) [1,2,3]. This inactive form is the main metabolite circulating in the blood and is also used for the classification of vitamin D status [1,2,3].

Predominantly in the kidneys, 25(OH)D is further hydroxylated to its most active form, 1,25-dihydroxy-vitamin D (1,25(OH)2D), by the enzyme, 1-α-hydroxylase. Since 1-α-hydroxylase is also found to be active in extra-renal tissues throughout the body [13], this gives rise to the assumption that vitamin D is playing a widespread role for overall health, including, beyond the musculoskeletal system, other tissues, such as the heart and the vessels.

### 1.2. Classification of Vitamin D Deficiency

Vitamin D status is classified according to 25(OH)D levels in the blood, and its half-life is approximately two to four weeks. There exists no clear consensus on the definition of vitamin D deficiency and vitamin D sufficiency. While the Institute of Medicine (IOM) report classifies vitamin D deficiency according to 25(OH)D levels below 12 ng/mL (multiply by 2.496 to convert ng/mL to nmol/L) and 20 ng/mL as sufficient, the Endocrine Society Guidelines suggest that 25(OH)D levels <20 ng/mL are deficient and levels of 30 ng/mL are sufficient [14,15,16]. These classifications are mainly based on bone related outcomes, since available data are still insufficient to give recommendations related to CVD or other chronic diseases.

### 1.3. Prevalence of Vitamin D Deficiency

Vitamin D insufficiency and deficiency are highly prevalent; this is very well reflected by the fact that more than half of the population worldwide has levels below 30 ng/mL [16,17]. Different factors, such as increased age, female sex, darker skin pigmentation, reduced sun exposure, as well as seasonal variation and distance from the equator are risk factors for vitamin D deficiency and must be considered. The increasing prevalence of low levels of vitamin D is mainly explainable by changes in lifestyle, reduced sun exposure and, to some extent, by air pollution [18]. It should, however, be acknowledged that previous inter-assay and inter-laboratory comparisons of 25(OH)D levels showed significant variability of the reported values. This, in turn, points to the need for standardization of 25(OH)D measurements and warrants caution when comparing 25(OH)D levels and their cut-offs derived from different studies [19,20,21].

## 2. Vitamin D and Cardiovascular Risk Factors

### 2.1. Arterial Hypertension

Vitamin D deficiency has been associated with higher blood pressure levels, which was already shown in most, but not all, prospective studies, as well as meta-analyses of observational studies [9,22,23]. While these observational data support an association between vitamin D status and blood pressure, it must also be acknowledged that residual confounding cannot be excluded. In addition, the reported variations in blood pressure explained by differences in vitamin D status were often relatively small and, thus, of questionable clinical relevance. Possible mechanisms for this association of vitamin D and blood pressure include the inverse association of vitamin D levels with the renin-angiotensin-aldosterone system (RAAS) activity, the effect of improving endothelial function and the prevention of secondary hyperparathyroidism [24,25,26,27]. In this context, it should be noted that high parathyroid hormone (PTH) levels are a hallmark of vitamin D deficiency and are known to be associated with myocardial hypertrophy and higher blood pressure levels [28]. In addition, increasing evidence suggests that the mutual interplay between vitamin D, parathyroid hormone and aldosterone mediates cardiovascular damage independent of the RAAS [29,30].

A large meta-analysis assessing the association of baseline vitamin D status with the risk of hypertension was performed by Kunutsor *et al.* They included 11 prospective studies published between 2005 and 2012, which comprised a total of 283,537 participants and 55,816 cases of hypertension with a mean follow-up of nine years [31]. The authors reported on a significant inverse association of baseline circulating serum vitamin D levels with the risk of incident hypertension. In detail, the pooled relative risk (RR) was 0.70 (95% confidence intervall (CI) 0.57–0.86) when comparing the highest to the lowest tertile of baseline 25(OH)D levels, with no evidence of heterogeneity among the findings. When evaluating dose-response in five studies that reported RRs for vitamin D exposure, the authors found that the risk for hypertension was lowered by 12% per 10 ng/mL increment of 25(OH)D [31]. Although this was the largest meta-analysis performed giving strong evidence for a relationship of vitamin D and blood pressure (BP), data on causality are still insufficient and warrant further RCTs.

Within the last year, several RCTs were performed to evaluate the effect of vitamin D on blood pressure (BP) levels in various cohorts, showing different results [32,33,34]. Larsen *et al*. performed an RCT in 130 hypertensive patients who were supplemented with 3000 IU of vitamin D or placebo over 20 weeks during winter in Denmark. They found a non-significant reduction of BP in the results of 24-h ambulatory blood pressure monitoring (ABPM) (−3 mmHg, *p* = 0.26/−1 mmHg, *p* = 0.18). Interestingly, when only vitamin D-insufficient patients were analyzed, with 25(OH)D levels below 32 ng/mL, (*n* = 92), systolic and diastolic BP levels in 24-h ABPM were significantly lowered (−4 mmHg, *p* = 0.05/−3 mmHg, *p* = 0.01) in the therapy group compared to placebo [32]. This effect in hypertensive and vitamin D-deficient patients has also been seen in a study by Forman *et al*., who performed an RCT in black Americans, who are known to be at a very high risk of both vitamin D deficiency and hypertension [33]. They included 283 participants who were allocated to either 1000, 2000 or 4000 IU of vitamin D or placebo over three months. They were able to show that supplementation of vitamin D in an unselected population of blacks lead to a reduction of 0.2 mmHg of systolic BP for each increase of 1 ng/mL of vitamin D over three months (*p* = 0.02) [33].

These results indicate an effect on BP of vitamin D, particularly in hypertensive, vitamin D-insufficient/deficient patients, rather than in a normotensive population with normal serum vitamin D levels. This should be considered for the design of future RCTs.

### 2.2. Obesity

Obesity is closely associated with vitamin D deficiency [35]. It had been hypothesized that this may be due to vitamin D deposition in adipose tissue, resulting in lower circulating 25(OH)D levels in the blood [36]. Others hypothesized a causal relationship of vitamin D deficiency leading to obesity [37]. To solve this research question, Vimaleswaran *et al.* performed a bi-directional Mendelian Randomization study and showed a one-directional causal relationship, indicating that obesity leads to lower vitamin D levels and not the other way around [38]. In that investigation, they included 21 cohorts, comprising a total number of 42,024 patients. They analyzed 12 established single nucleotide polymorphisms (SNPs) related to body mass index (BMI) and four typical vitamin D-related SNPs to perform this bi-directional Mendelian Randomization study. They could show that each unit increase of BMI was associated with a 1.15% decrease of 25(OH)D after adjustments for typical confounders. The authors concluded that obesity can be regarded as a causal risk factor for vitamin D deficiency, accounting for approximately one third of vitamin D deficiency [38]. On the other hand, genetically determined 25(OH)D levels were not significantly related to BMI. These findings suggest that the link between obesity and vitamin D deficiency is only driven by the fact that a higher BMI lowers 25(OH)D levels. By contrast, there seems to be no significant effect of vitamin D status on obesity. While these findings are important for our understanding on the causality regarding the association between vitamin D and obesity, there are several unanswered questions surrounding this topic related, e.g., to the bioavailability of vitamin D stored in adipose tissue.

### 2.3. Glucose Metabolism and Diabetes Mellitus Type 2

In observational and prospective studies, low vitamin D levels have largely been associated with disturbances in glucose metabolism, as well as higher risk of developing diabetes in the future, although some authors have reported on conflicting results [5,39,40,41,42]. It should also be kept in mind that vitamin D deficiency in diabetic patients may partly be a consequence of reduced physical activity and consecutive obesity, as well as limited sun exposure. Therefore, residual confounding in observational studies due to the close link of obesity with both vitamin D deficiency and glucose intolerance cannot be ruled out with certainty [35,43]. On the other hand, we must also consider that reverse causality may exist, since there are data suggesting that an inflammatory insult might decrease 25(OH)D levels [44].

There are, however, several possible mechanisms that could explain the association of vitamin D deficiency with disturbances in glucose homeostasis and diabetes mellitus. VDR, as well as 1-α-hydroxylase are expressed in pancreatic beta cells, indicating a potential role of vitamin D on beta cell function [2,45]. It has also been hypothesized that calcium, which is crucial for insulin synthesis and secretion, could play a role, since it is mainly regulated by vitamin D [46]. Another possible pathway could be vitamin D-induced stimulation of osteocalcin, which may improve insulin sensitivity [47].

Randomized trials, on the other hand, have largely failed to show clear beneficial effects of vitamin D supplementation on improving glycaemia or insulin resistance [48,49]. Addressing this issue, Davidson *et al.* conducted an RCT in individuals with prediabetes and hypovitaminosis D [50]. Study participants were allocated to high dose vitamin D therapy (mean weekly dose of 88,865 IU) *vs.* placebo [50]. No difference regarding plasma glucose parameters, insulin secretion and sensitivity or development of diabetes in the therapy group compared to placebo administration was found after one year [50].

Hence, although some preliminary data suggested a relevant effect of vitamin D on glucose homeostasis, the currently available literature on vitamin D does not support the notion that vitamin D supplementation is useful for the prevention and/or treatment of diabetes mellitus. Further RCTs are, however, urgently needed before drawing final conclusions on the relationship between vitamin D and diabetes.

### 2.4. Lipids

Some observational studies indicate an association of vitamin D deficiency with lower high density lipoprotein (HDL) and higher triglycerides, as well as higher apolipoprotein E levels [51,52]. Towards this, a large prospective evaluation of vitamin D levels and blood lipids showed a significant association of lower vitamin D levels with hypercholesterinemia [53]. However, it should be acknowledged that the results on vitamin D and blood lipids are inconsistent and could be confounded by the above mentioned link of vitamin D and obesity [38].

Recent clinical studies that have evaluated the effect of vitamin D supplementation on blood lipids in some RCTs yielded conflicting evidence. They showed rather inconsistent findings with the majority of the studies reporting on no significant effect on blood lipids when vitamin D supplementation was compared to placebo [33,54,55,56].

These recent results aggravate the decision on a causal relationship of vitamin D deficiency and an unfavorable lipid profile. Nevertheless, no final conclusion can be drawn since large, well-designed RCTs are still missing in this field. In addition, we should also consider that publication bias, *i.e.*, unpublished results showing no effects of vitamin D, might be a problem.

### 2.5. Chronic Kidney Disease

Vitamin D levels in patients with chronic kidney disease (CKD) are significantly lower compared to the general population. For example, a high prevalence of vitamin D deficiency with values of below 20 ng/mL in more than 70% was seen in dialysis patients [57]. This may be due to the fact that these patients have a reduced sun exposure, due to a higher prevalence of co-morbidities. Moreover, it has also been suggested that CKD patients have an impaired vitamin D synthesis in the skin.

Several epidemiological studies have shown that lower 25(OH)D levels were associated with albuminuria and/or progression of renal failure. Moreover, vitamin D deficiency has been identified as an independent risk factor for higher mortality in patients suffering from CKD, which can mainly be attributed to cardiovascular deaths [58,59,60,61]. Apart from low 25(OH)D, also low 1,25(OH)2D was associated with higher mortality rates in most observational studies among CKD patients [62,63].

Particular attention is paid to vitamin D in the field of nephrology, because the classic and broadly known effect of vitamin D supplementation is the reduction of PTH levels. This is of high clinical relevance, since PTH itself is an independent cardiovascular risk factor [64], and secondary hyperparathyroidism is very common in CKD patients. This therapeutic effect of PTH lowering is achieved with both active (1,25(OH)2D) and natural (25(OH)D) vitamin D supplementation, although stronger when supplementing with 1,25(OH)2D [65].

Addressing the role of active vitamin D treatment in CKD, Duranton *et al*. conducted a systematic review and meta-analysis of seven prospective and seven retrospective observational trials in CKD patients treated with 1,25(OH)2D or different active vitamin D analogues. The authors found a significant reduction of all-cause mortality (RR 0.73; 95% CI 0.65–0.82) and even 37% reduction of cardiovascular mortality (RR 0.63; 95% CI 0.44–0.92) in patients on active vitamin D treatment [66].

While these data suggest beneficial effects of vitamin D treatment in CKD patients, it must also be pointed out that no major vitamin D RCTs have evaluated hard clinical endpoints in CKD patients yet; though, meta-analyses of randomized trials performed in older study populations, of which a great part showed impaired kidney function, showed a reduction of fractures and all-cause mortality by vitamin D supplementation [67,68].

### 2.6. Endothelial Dysfunction/Atherosclerosis

Since the VDR is also expressed in the vasculature, it is tempting to hypothesize that vitamin D might also protect against vascular diseases, including atherosclerosis and endothelial dysfunction [27]. According to experimental studies, some putative vasculoprotective actions of vitamin D may be mediated by increasing nitric oxide (NO) production, inhibiting macrophage to foam cell formation or reducing the expression of adhesion molecules in endothelial cells [69,70,71]. This is in line with reports from cross-sectional observational studies, which showed that lower vitamin D levels are associated with endothelial dysfunction, as well as increased arterial stiffness [6,27].

Clinical data from RCTs addressing vitamin D effects on vascular diseases are sparse and revealed inconsistent results. Promising results were, however, published on vitamin D and endothelial function with some, but not all RCTs showing that vitamin D may improve endothelial function [72,73,74].

## 3. Observational Studies on Vitamin D and Cardiovascular Events

Already in 1981, Scragg found an inverse relationship of cardiovascular mortality and UVB radiation [8]. Since then, several, but not all, observational studies that have been published indicated that low vitamin D levels are associated with higher incidence of cardiovascular events and mortality [10,66,75,76,77]. Even asymptomatic coronary artery disease was associated with lower vitamin D levels in high risk type 2 diabetic patients (adjusted odds ratio (OR) 2.9, 95% CI 1.02–7.66), as observed in a recent observational study [78].

Vitamin D deficiency has been associated with an increased risk of myocardial infarction (MI), and a significant inverse relationship of 25(OH)D levels and matrix-metalloproteinase-9 (MMP-9), a marker for myocardial remodeling after acute MI, has been documented [79,80]. Vitamin D levels also seem to predict the risk of adverse events after acute myocardial events and cardiac surgery, indicating higher risk for patients with lower vitamin D levels, as reported in recent publications [81,82].

Data from prospective observational studies suggest that low vitamin D levels are a risk factor for the occurrence of strokes [83,84,85]. Chowdhury *et al.* showed in a meta-analysis of seven studies, including 47,809 individuals and 926 cerebrovascular events that, under consideration of established cardiovascular risk factors, the risk for cerebrovascular disease was significantly lower in subjects with high 25(OH)D levels compared to those with insufficient vitamin D status [84]. Another meta-analysis reported on similar results when comparing low *versus* high vitamin D levels, with an RR for strokes of 1.52 (95% CI 1.20–1.85) in the lowest *versus* the highest 25(OH)D group [83].

In the currently largest meta-analysis on circulating 25(OH)D levels and risk of CVD, Wang *et al*. could show an adjusted pooled RR of 1.52 (95% CI 1.30–1.77) for total CVD when comparing the lowest to the highest categories of baseline circulating 25(OH)D concentration [12]. The authors investigated 19 studies, including 65,994 patients and 6123 CVD cases. The increment in CVD risk across decreasing 25(OH)D levels was generally linear over the range of 25(OH)D levels from 20 to 60 nmol/L, with a marginally significant pooled RR of 1.03 per decrement of 25 nmol/L of 25(OH)D [12]. It should, however, be noted that not all single studies reported on a significant association between low 25(OH)D levels and increased risk of CVD [86].

When reviewing these above mentioned meta-analyses, it has to be kept in mind that these observations could also be influenced by confounding factors, such as reduced mobility and physical activity in chronically ill patients, therefore leading to reduced sunlight exposure and lower vitamin D levels. Other confounders, such as increased age or higher rate of obesity, as well as PTH, renin, calcium and phosphorus, cannot be ruled out with certainty, although they are included as possible confounders in most trial analyses [25,87].

## 4. Vitamin D Supplementation and Cardiovascular Disease

There are only a few RCTs that have evaluated cardiovascular outcomes, and since all large previous RCTs were designed to study vitamin D effects on bone health, most of them were neither primarily designed nor statistically powered to assess vitamin D effects on CVD. Some small RCTs reported on mixed results of vitamin D supplementation on cardiovascular events [33,88], although it bears mentioning that some meta-analyses of these RCTs have found non-significant trends for reduced CV-events in patients receiving vitamin D supplementation compared to placebo [89,90]. Of note, vitamin D supplementation was very often combined with calcium intake, making it hard to interpret the RCT results, especially since calcium intake may be associated with increased cardiovascular risk, as suggested in a previous meta-analysis [91].

Since CVD is globally the leading cause of death, it is of special interest that in a Cochrane review and meta-analysis on randomized controlled trials from 2011, Bjelakovic *et al*. could show that vitamin D supplementation leads to a moderate, but statistically significant, reduction of total mortality rate (RR 0.94, 95% CI 0.91–0.98) compared to placebo [68]. The authors calculated that 161 individuals have to be treated to prevent one additional death [68]. This result by Bjelakovic *et al*. was mainly derived from RCTs in older individuals and is in line with several observational studies suggesting that vitamin D deficiency is a risk factor for mortality, particularly in the aging population [92]. The findings by Bjelakovic should, however, be interpreted with caution, since competing risks in elderly people might have an impact on the results. In addition, it must also be underlined that the study by Sanders *et al.* showed that high-dose vitamin D supplementation caused an increased risk of falls and fractures [93].

## 5. Discussion and Future Outlook

Based on systematic reviews and meta-analyses of the currently available literature, it can be concluded that vitamin D deficiency is an independent cardiovascular risk factor that is associated with increased risk of cardiovascular events. However, it is largely unclear whether these associations are of causal nature. While it seems plausible that vitamin D deficiency can be considered a surrogate marker for poorer health status, most notably observed in patients with chronic diseases, including cardiovascular risk factors and CVD, it remains to be proven whether vitamin D itself can directly impact on cardiovascular outcomes [94].

Several RCTs in the past failed to prove a causal relationship between vitamin D repletion and reduction of CV risk factors and CVD. This could hypothetically be attributed to small sample sizes or inappropriate study designs, since most trials were initially designed for clinical endpoints other than cardiovascular events [33,88,89,90]. Independent of these conflicting results, it is not entirely clear whether vitamin D supplementation exerts significant beneficial effects on healthy population, in addition to multimorbid patients. Of note, most trials have shown that beneficial effects of vitamin D supplementation are frequently identified in patients with very low 25(OH)D levels, and these patients seem to be at the highest risk for CVD [11,12,31,32,73,95]. On the other hand, it is not entirely clear whether vitamin D supplementation has significant beneficial effects in healthy population, as well, or is only meaningful in vitamin D deficient, chronically ill patients.

While existing data are insufficient to draw final conclusions on the effect of vitamin D supplementation on cardiovascular outcomes, several large interventional trials, designed to evaluate the effect of vitamin D supplementation on different CVD as primary endpoints in chronically ill, as well as general populations, have just started. These include a study in 1000 heart failure patients in Germany, the EVITA study by Zittermann *et al.*, the Vitamin D Assessment Study (ViDA) in New Zealand among over 5000 older individuals conducted by Scragg *et al*. and the *VIT*amin D and Omeg*A*-3 Tria*L* VITAL trial, evaluating cardiovascular and cancer mortality in over 20,000 older subjects in the USA without cancer or CVD at baseline [96,97,98]. The large sample size of all these studies and a rather long time of intervention seem promising to gain final conclusions, especially regarding vitamin D effects on cardiovascular events and mortality in the general population. Results are expected in the next few years (2017–2020), but it is still open whether the findings of these RCTs will give all the final answers on the question of whether vitamin D is useful for the prevention and treatment of CVD. It has to be kept in mind that patients included in, for example, the VITAL study are not screened for vitamin D deficiency prior to inclusion, and also, an additional amount of up to 800 IU of vitamin D supplementation is also allowed in the placebo group [96]. This could lead to relatively high vitamin D levels in the placebo group, which could mask a possible beneficial effect of supplementation in individuals with very low vitamin D status [77]. Another possible problem with the VITAL study is the vitamin D food fortification in the US. Therefore, we can hope to gain new insights on the association of vitamin D and CVD, but cannot be sure to receive definite answers to be able to recommend vitamin D supplementation as a preventive or therapeutic method in this context.

## 6. Conclusions

At present, we can conclude that vitamin D deficiency is an independent cardiovascular risk factor, but whether vitamin D supplementation can significantly improve cardiovascular outcomes is still largely unknown.
